# The role of circular RNAs in the pathophysiology of oral squamous cell carcinoma

**DOI:** 10.1016/j.ncrna.2022.11.004

**Published:** 2022-11-22

**Authors:** Albert Sufianov, Sema Begliarzade, Valentin Kudriashov, Aferin Beilerli, Tatiana Ilyasova, Yanchao Liang, Ozal Beylerli

**Affiliations:** a Educational and Scientific Institute of Neurosurgery, Рeoples’ Friendship University of Russia (RUDN University), Moscow, Russia; b Department of Neurosurgery, Sechenov First Moscow State Medical University (Sechenov University), Moscow, Russia; c Republican Clinical Perinatal Center, Ufa, Republic of Bashkortostan, 450106, Russia; d Gastric Cancer Center, West China Hospital of Sichuan University, China; e Department of Obstetrics and Gynecology, Tyumen State Medical University, 54 Odesskaya Street, 625023, Tyumen, Russia; f Department of Internal Diseases, Bashkir State Medical University, Ufa, Republic of Bashkortostan, 450008, Russia; g Department of Neurosurgery, The First Affiliated Hospital of Harbin Medical University, Harbin, 150001, China

**Keywords:** Non-coding RNAs, Circular RNAs, Biology function, Oral squamous cell carcinoma, Biomarker

## Abstract

Circular RNAs are non-coding RNAs that widely exist in eukaryotes. The research progress of its generation mechanism and biological function show that circular RNAs may be used in the development of tumors, neurological diseases, cardiovascular diseases. They play an important role in the occurrence and development of diseases and has a potential to be used as a disease marker. Oral squamous cell carcinoma is one of the most common malignant tumors in oral surgery. It is difficult to treat, easy to metastasize, and has a poor prognosis. Due to its unclear mechanism, blocking oral squamous cell carcinoma at the genetic level cannot be achieved. The research progress of circular RNA in the field of oral squamous cell carcinoma will bring new ideas for the biological treatment of oral squamous cell carcinoma. This review summarizes the circRNAs mechanism, the biological function and the research progress in the development of tumors, especially oral squamous cell carcinoma.

## 1 Introduction

Circular RNAs (circRNAs) are a new member of the long non-coding RNAs (lncRNAs) family [1]. Unlike linear RNA, this RNA does not have the characteristics of 5′methylguanosine cap and 3′polyadenylation tail, but a covalent closed-loop structure, so circular RNA is not effective for ribonucleases (Rnases), what makes they more sensitive and more stable than linear RNA [2]. According to different sources, circular RNA can be divided into exonic circular RNA (exonic circRNA), intronic circular RNA (intronic circRNA), exon-intron circular RNA (exon-intron circRNA), and tRNA-derived circular RNA (TriRNA) [[3], [4], [5], [6]]. Research has shown that circular RNAs are widely involved in the pathogenesis of various diseases, and their complex and diverse functions, unique abundance, breadth, stability and tissue specificity play an important role in the pathological process and become a biological star in the field of science [7].

Oral squamous cell carcinoma (OSCC) is the 11th most common cancer in the world [8]. OSCC has the characteristics of local invasiveness, high recurrence, and easy metastasis. The 5-year survival rate is lower than performed in other solid tumors, which is only 50–60% [9]. However, the molecular mechanism of OSCC is still unclear, and there are no highly sensitive and specific biomarkers as monitoring indicators for the early diagnosis, treatment and prognosis of OSCC. Previous literature has shown that circRNAs are associated with the malignant progression of OSCC, suggesting that circRNAs have potential functions as markers for early diagnosis and biological treatment of OSCC. This article briefly introduces the characteristics and the generation process of circular RNAs, and discusses their research progress in tumors, especially OSCC, from their biological functions.

## 2 The mechanism of circular RNAs formation

In eukaryotes, canonical splicing is formed by the spliceosome machinery, which removes introns and joins exons into one linear RNA transcript [10]. Most circular RNAs are derived from exons. The whole process can be divided into two parts: 1) the upstream intron of one or more exon pairs is connected with the downstream intron; 2) the 2′-hydroxyl of the upstream intron is connected with the 5′-hydroxyl of the downstream intron The phosphate group reacts, and then the 3′-hydroxyl group of the 3′-exon reacts with the 5′-phosphate of the 5′-exon, finally forming a circular RNA [11]. ALU sequences in introns can also interact, promoting back splicing [12]. Some RNA-binding proteins act as regulators in this process. For example, the RNA-binding protein MBL joins proximal introns and promotes the production of circular RNAs; while the RNA editing enzyme ADAR1 inhibits the production of circular RNAs [13,14]. In addition, a lasso-driven circularization is another mechanism for the generation of circular RNAs, upstream splice acceptors and downstream donors are brought close to each other due to the presence of RNA lasso, while exons are formed by 5′-3′ phosphodimers ester linkage [15].

## 3 Biological functions of circular RNAs

**Fig. 1:**
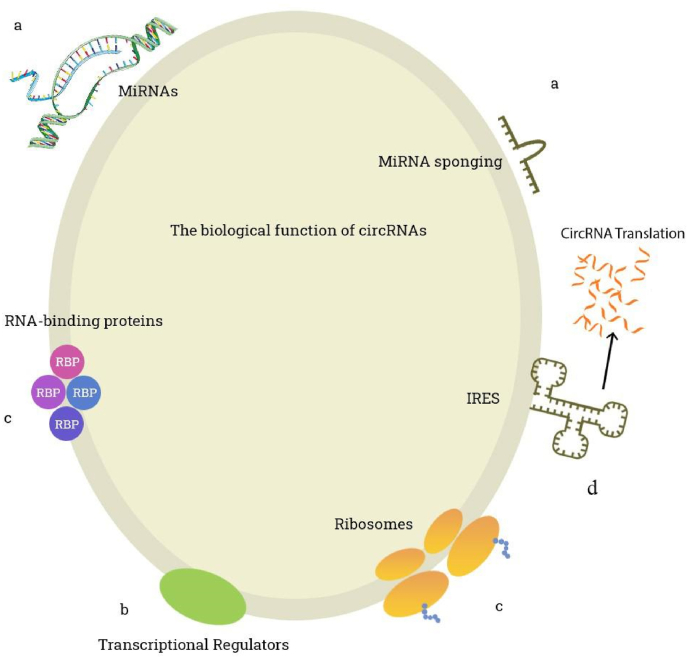
The biological functions of circRNAs.
b) Regulation of linear RNA transcription. Circular RNA can promote or inhibit the transcription of linear RNA and is a key regulator of alternative splicing or transcription. Circular RNAs formed by exons constitute a vast majority of known circular RNAs, and their generation process has a competitive impact on conventional splicing.
c) Sponging on proteins. Circular RNAs can also adsorb RNA binding proteins (RNA binding proteins, RBPs) to regulate protein levels. For example, circ-MBL found in flies and humans can bind to MBL proteins at multiple binding sites [7]. An increasing number of studies have shown that some circular RNAs are also able to interact with proteins, thereby affecting the behavior of cells [18]. For example, circFOXO3, a circular RNA that has received the most extensive attention, has been shown to be an adaptor linking p21 and cyclindependent kinases 2 (CDK2). circFOXO3 promotes the release of CDK2 from p21, which can phosphorylate cyclin A and cyclin E, thereby promoting cell division and proliferation. High expression of circFOXO3 can also promote the interaction between p53 and MDM2, thereby accelerating the degradation of p53 [19].
d) Regulation of protein translation. The translation is performed by ribosomes and involves initiation, elongation, termination and ribosome recycling [20]. The Initiation on eukaryotic mRNAs involves scanning by 43S preinitiation complexes from the 5′ cap-proximal point of attachment to the initiation codon, followed by ribosomal subunit joining and factor displacement [21]. Lacking the 5′-cap and 3′-tail, circRNA can only adopt cap-independent manners. In addition to previously described m6A-mediated translation [22], artificial and endogenous circRNAs containing an internal ribosome entry site (IRES) that directly recruits ribosomes [23], can also be translated. These two approaches may be coupled with each other. For example, m6A improves the efficiency of IRES-mediated translation of circZNF609 [24]. Additionally, circRNA with an infinite ORF undergoes rolling circle amplification in an IRES-independent manner, leading to a hundred-fold higher productivity than linear transcript [25]. Peptides encoded by circRNAs are generally truncated and their functions are mostly analogous to the full-length protein counterparts (circFBXW7-185aa [26]). However, some proteins originating from circRNAs exert functions are independent of or even opposed to those of their host gene products (circFNDC3B-218aa) [27]. These results broaden the range of human proteome. However, the regulatory mechanisms of circRNA translation and the processes of elongation and termination are still not completely understood.

Circular RNAs have diverse functions as non-coding RNAs, which are mainly divided into the following four aspects:

a) Sponging of miRNAs. miRNAs are considered to be endogenous non-coding RNAs, which can inhibit downstream gene expression, thereby inhibiting protein synthesis. More and more studies have found that circular RNAs with complementary sequences can sponge-like adsorb miRNAs and inhibit their downstream gene expression [7]. For example, cirs-7, also known as CDR1as, is a circular RNA containing more than 70 miR-7 binding sites, which can bind to miR-7 and act on its downstream mRNA [5,16]. This molecular pathway axis is widely expressed in diseases such as astrocytoma and lung cancer (Fig. 1) [17].

## 4 Circular RNAs and tumors

Studies have shown that circular RNAs play an important role in the occurrence and development of solid tumors and hematological malignancies, especially through their biological functions as miRNA sponges. Circular RNAs are involved in several characteristic processes of tumorigenesis and development, such as the evasion of growth suppressors, maintenance of proliferative signals, the evasion of cells’ death and senescence, and the ability to promote angiogenesis and activate the invasion and metastasis. In addition, the abnormal expression, the tissue specificity, the diversity and the stability of circRNAs in tumor cells make them have a great potential as tumor markers. In liver cancer, researchers have found that the expression of hsa_circ_0005075 in 60 groups of liver cancer tissues was significantly different from normal tissues, indicating that hsa_circ_0005075 is a potential biomarker [28]. In addition, the expression of hsa_circ_0001649 is also different, and is closely related to the size of HCC tumor and the occurrence of tumor thrombus, while circZFR, circFUT8 and circIPO11 have been proved to be useful for the identification of HCC specimens [29,30]. For lung cancer, both clinical cohort studies and cell-level studies have found that circRNAITCH plays an inhibitory role in lung cancer. Abnormal regulation of circRNA-ITCH can enhance the expression of its parent tumor suppressor gene ITCH by sponging miR-7 and miR-214, thereby regulating the proliferation of cancer cells [31]. circ-001569 can sponge-like adsorb miR-145 and upregulate the expression of its downstream genes such as E2F5, BAG4 and FMN12, resulting in an increase in G2/M phase cells and a decrease in tumor cell apoptosis [32]. In addition, in non-solid tumors, Salzman et al. serendipitously found genes producing hundreds of circular RNAs in acute myeloid leukemia, and these circular RNAs were also detected in HeLa cells [33]. Another study has confirmed that chemotherapy can inhibit the abnormal expression of hsa_circ_0004277 in patients with acute myeloid leukemia [34]. In addition to the above tumors, studies have also found that some circRNAs are associated with gastric cancer, breast cancer, colon cancer, bladder cancer, ovarian tumors, and skin squamous cells [[35], [36], [37], [38], [39], [40], [41], [42]]. It is closely related to various cancers such as epithelial carcinoma [43].

## 5 Circular RNAs and OSCC

### 5.1 Research status of circular RNAs in OSCC

The application of high-throughput sequencing has allowed more and more OSCC-specifically expressed circular RNAs to be screened. Studies have screened circRNAs with significant differences between OSCC tissue and paracancerous tissue by circRNA chip technology, including 155 circRNAs with a relative expression ratio of more than 1.5 times. Circular RNA hsa_circ_0001874 was shown to be closely related to a tumor's clinical stage and a degree of differentiation, and its expression in low differentiated OSCC tissues has been significantly higher than in moderately and highly differentiated OSCC tissues [44]. Through the prediction of circular RNA target gene analysis software, it is inferred that miR-103A-3P, miR-107, miR-593–5p, miR-661 and miR-662 may be the sponge targets of hsa_circ_0001874, but the specific functions are still unknown. Similar studies have also included exploring the expression profiles of circRNAs in tongue squamous cell carcinoma tissues and normal paracancerous tissues. High-throughput sequencing was performed in 4 cases of cancer foci and 4 cases of adjacent tissues. A total of 17 171 circRNAs with differences were screened, among which 15 circRNAs had a 50-fold difference [45]. The upper circular RNA hsa-circ-0033 967 with a differential fold of 116.31 is predicted to have 163 potential binding sites for hsa-miR-608, and hsamiR-608 has been confirmed to play a tumor suppressor role in liver cancer glioma in recent years [46]. This finding suggested that hsa-circ-0033 967 could block the tumor suppressor effect of hsa-miR-608 through a sponge action, thereby promoting the occurrence and the development of tongue cancer. Some circular RNAs have been shown to have unique signaling pathways in the malignant process of OSCC, and have clinical application value. Cyclin-dependent kinases 6 (CDK6) promote tumorigenesis at some specific stages by regulating transcriptional responses. Wang et al. established a cell apoptosis model based on three oral squamous cell carcinoma cell lines CAL-7, SCC-9, and SCC-25 [47]. Compared with the control group, it was found that there were differences in the expression of 628 circRNAs, among which the circRNA DOCK1. It is one of the circular RNAs whose expression is significantly decreased in the apoptosis model. Real-time quantitative polymerase chain reaction found that this circular RNA is also highly expressed in OSCC cell lines and tissues. In this low-expression model of circular RNA, the expression level of miR-193a-5p, which plays an important role in various apoptosis pathways, increased, and at the same time, the apoptosis rate increased to varying degrees. Further experiments proved that miR-193a-5p could bind to the mRNA of baculoviral IAP repeat containing (BIRC) to reduce the content of BIRC in cells and increase the apoptosis rate. Chen X. et al. have found that circ_0014 359 was highly expressed in the OSCC tissues and cell lines compared to the normal controls and that expression was associated with the survival of patients. For the OSCC cell lines, circ_0014 359 knock down induced apoptosis and inhibited migration, invasion, and epithelial-mesenchymal transition of OSCC cells. In vivo, silencing the circ_0014 359 blocked the growth of OSCC tumors. The circ_0014 359 can directly interact with the micro-RNA-149 (miR-149) [48]. The existence of −196a-5p/BIRC3 signaling pathway-circRNA DOCK1 can indirectly affect the apoptosis of OSCC by regulating the expression of the protein BIRC, which reflects the potential of circRNA DOCK1 as a diagnostic biomarker and therapeutic target for OSCC. Another study has found that another circular RNA may also assist in the diagnosis of OSCC [49]. Cui L. et al. have found that CircCDR1as acted as an oncogene in OSCC progression through elevating SLC7A11 by targeting miR-876–5p [50]. The expression of hsa_circ_1001242 was significantly increased in 4 oral squamous cell carcinoma cell lines SCC-9, SCC-15, SCC25, and CAL-27, and the expression of hsa_circ_1001242 was significantly increased. Levels and tumor size were negatively correlated with T stage. Similarly, the expressions of hsa_circ_0001874 and hsa_circ_0001971 in saliva were significantly increased in OSCC samples, and they have the potential to be used as OSCC tumor markers to judge the degree of tumor development, because their contents are related to the TNM stage of OSCC, and the former is related to the tumor grade. The hsa_circ_0001874 had a higher expression level in the more malignant samples. Not only that, OSCC and oral leukoplakia can also be distinguished by comparing the differences in the expression levels of these two circular RNAs, and surgery will also reduce the content of the two circular RNAs in saliva [51]. Not all circRNAs in OSCC are reported below. The only circRNA forming the circRNA-mRNA regulatory axis is described, along with a description of the role in other forms of cancer to understand the diversity of function. Table 1 provides a succinct description of the regulatory network and expression levels in OSCC tissues.

**Table 1:** A Short display of the above described circRNA-miRNA pathway regulatory axis.

circRNA	Expression IN OSCC	Sponged miRNA	Regulatory Axis	Reference
ciRS-7	Upregulated	miR-671–5p	ciRS-7**/**miR-671–5p/CDR1/AKT/ERK_1/2_/mTOR/ROS	47
circPVT1	Upregulated	miRNA-125b	circPVT1/miRNA-125b/STAT3	52
circHIPK3	Upregulated	miR-124	circHIPK3/miR-124/ITGB1	53
circMDM2	Upregulated	miR-532–3p	circMDM2/miR-532–3p/HK2	54
circPKD2	Downregulated	miR-204–3p	circPKD2/miR-204–3p/APC2/WNT/β-catenin/p-AKT/p-ERK1/2	55
circDOCK1	Upregulated	miR-196a-5p	circDOCK1/miR-196a-5p/BIRC3	56
circATRNL1	Downregulated	miR-23a-3p	circALTR1/miR-23a-3p/PTEN/AKT/P13K/ATM/ATR/P53	57
hsa_circRNA_100 533	Downregulated	miR-933	hsa_circRNA_100533/miR-933/GNAS	58
hsa_circRNA_100 290	Upregulated	miR-378a	hsa_circRNA_100 290/miR-378a/GLUT1	59
hsa_circ_000140	Downregulated	miR-31	hsa_circ_000140/miR-31/LATS2	60
hsa_circ_0001971	Upregulated	miR-104, miR-204	hsa_circ_0001971/miR-104/miR-204/P13K/AKT/FoxO3a	61
hsa_circ_0008309	NC: Variation but significantly showed downregulation	miR-136–5P, miR-382–5P	hsa_circ_0008309/miR-136–5P/miR-382–5P/ATX1	62

### 5.2 Application value of circular RNAs in OSCC

**Fig. 2:**
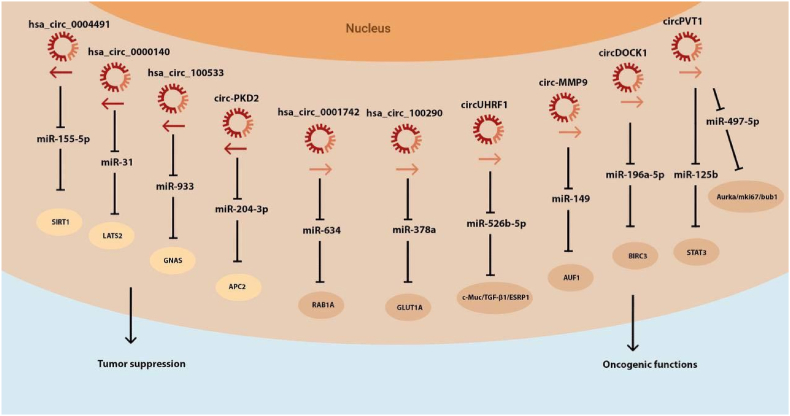
Schematic diagram of the circRNA-miRNA networks in OSCC.

Nowadays, some progress has been made in early diagnosis, surgical methods, and radiotherapy and chemotherapy for OSCC. However, the early symptoms of OSCC are not typical, and tissue biopsy is often required for final diagnosis. There is still a lack of rapid, accurate and non-invasive early diagnosis in clinical work. Circular RNAs are abundant, widespread, conserved, and can be stably expressed in saliva, blood, urine, and exocrine vesicles, and their expression in tissues and organs is stage-specific. Since OSCC occurs in the mouth, the test of saliva samples may become an early screening and a diagnosis method for OSCC high-risk markers, and the operation is simple and does not require invasive procedures to obtain samples. Several studies have shown that microRNAs (miRNAs) and long noncoding RNAs (lncRNAs) in saliva can be used as early diagnostic markers for OSCC [63,64] [Fig. 2]. Compared with long non-coding RNAs, circular RNAs are less sensitive to RNases due to their closed loop structures, and the half-life of circular RNAs is often higher than 48 h, while the half-life of microRNAs is only 10 h on average. Therefore, circular RNAs are more suitable as markers [65]. Literature has shown that there are OSCC-specific circular RNAs in saliva. As mentioned above, the content of has-circ-0001874 and has-circ-0001971 in the saliva of OSCC patients are significantly increased. Specific circular RNAs are used as the strong evidence for the diagnosis of OSCC [51]. Similarly, circRNAs can also be used as prognostic markers for OSCC cyclic, and these OSCC-specific circRNAs can be used as monitoring indicators for regular follow-up of patients after surgery or medical treatment [66]. Research shows that some circRNAs can regulate the biological behavior of tumor cells through intracellular signaling pathways, so circRNAs are considered to be one of the future molecular biology therapeutic targets for OSCC in case circRNAs can be interfered by drugs and inhibit the proliferation of OSCC cells and promote apoptosis [67]. Nowadays, the most important method to interfere with the expression of circRNAs is to construct a targeted small interfering RNA (siRNA) of a specific type of circRNA through a database query, and to introduce it into OSCC cells, which can reach the level equivalent to the gene encoding the circRNA, thereby hindering the further development of cancer cells or cancerous tissue by the knockout effect. Research found that the introduction of circRNA-100290 targeting siRNA into OSCC cell lines inhibited the proliferation of OSCC cell lines in vitro and in vivo by regulating the circRNA-miR29 family-CDK6 pathway [25]. Similarly, Wang et al. constructed a low-expression model of this circRNA by introducing siRNA of circRNA DOCK1 and found that the apoptosis rate of CAL-7, SCC-9, and SCC-25 3 OSCC cell lines increased [68]. Using molecular biological methods to maintain the low expression state of circRNAs that promote the malignant development of OSCC has the potential to be used in clinical treatment.

On the contrary, the overexpression of certain circular RNAs has a certain inhibitory effect on the proliferation and development of cancer cells. It has become possible to synthesize circular RNAs with specific benign effects by artificial means and to transfect them into cells to achieve therapeutic effects. Some researchers have successfully synthesized a circular RNA-scRNA21 that can significantly inhibit the proliferation of cancer cells in vitro [69]. This circular RNA can bind miR-21 through sponge action when introduced into cells, thereby up-regulating the target inhibition of miR-21. The expression of tumor suppressor gene Daxx induces the apoptosis of gastric cancer cells. By increasing the intracellular content of circRNAs and then affecting the biological behavior of OSCC cells, it provides a new idea for the treatment of OSCC.

## 6 Conclusions

Although circular RNAs have high advantages as biomarkers for diagnosis, prognosis and treatment of OSCC, the current research results still have great limitations. For example, when analyzing the differentially expressed circRNAs in OSCC patients, the size of a sample and a selection of the samples will affect the final data, and the current research results cannot represent the situation of all OSCC patients. In summary, as a molecule that has just been discovered to have special biological functions, circular RNA has good specificity and stability, and its potential as a tumor marker has been demonstrated in gastric cancer, colon cancer, breast cancer and other cancers [[29], [30], [31], [32], [33], [34]]. OSCC is a cancer that is prone to metastasis and has a poor prognosis, and its mechanism of occurrence and development is still unclear. Revealing the occurrence and development of OSCC at the molecular level will certainly provide more information on the early diagnosis, treatment and prognosis of OSCC and become a more efficient and accurate method. Studies on the role of circRNAs in OSCC have also appeared one after another, providing new ideas for tumor diagnosis and prognosis. Although more and more circRNAs that are differentially expressed between tumor tissues and normal tissues have been discovered, only a small number of circRNAs have been elucidated for their precise roles in cellular and molecular mechanisms, and the mechanism of action of the vast majority of circRNAs is still uncertain, and there is still much unknown to be explored [[70], [71], [72], [73], [74], [75], [76]]. Moreover, before the role of circRNA as a tumor diagnostic marker transitions from a theory to a clinical application and becomes a simple, efficient and accurate laboratory examination method, a plenty of research is needed as a basis.

## Funding

This work was supported by the Bashkir State Medical University Strategic Academic Leadership Program (PRIORITY-2030).

## Author contributions

Albert Sufianov and Sema Begliarzade conceptualized and designed the study. All authors have participated in the acquisition, analysis and interpretation of the data. Valentin Kudriashov has drafted the manuscript. Tatiana Ilyasova, Yanchao Liang contributed to the critical revisions of the manuscript. All authors agreed on the journal to which the article would be submitted, gave the final approval for the version to be published, and agreed to be accountable for all aspects of the work.

## Declaration of competing interest

The authors declare they have no conflict of interest.
